# Society Proceedings

**Published:** 1912-06

**Authors:** 


### SOCIETY PROCEEDINGS

THE 7th Anti-Tuberculosis Congress was held in Rome, April, 1912. We are informed that Menciere of Rheims opposed the abuse of climatic treatment of osseous and articular tuberculosis, which he declared to be curable everywhere, by every practitioner who would give it proper attention. He laid great stress on iodoform ointment and "phenolization," in grave cases. The latter often prevents amputation and always cures without resection.

The Dental Society of the State of New York, on May 10, 1912, elected the following officers: President, C. F. Baylis, Oneonta; V. P., W. W. Smith, Rochester; Secretary, A. P. Burkhardt, Auburn; Treasurer, George H. Butler, Syracuse.

The Niagara Frontier Pure Water Conference, met in Buffalo, May 10, 1912. Hon. Wm. L. Mills of Tonawanda presided. He declared that a person contracting typhoid fever by drinking impure water had a cause of action against the municipality. Dr. E. H. Porter, State Health Commissioner, observed that it would be necessary to prove that the infecting water was actually obtained from the city supply. Some one in the discussion stated that a $10,000 verdict on such an action had actually been awarded in Minnesota. Concerted action along the Frontier was urged.

The annual meeting of the Steuben Co. Medical Society was held in Bath, May 14. Officers were elected as follows: President, John Conway, Hornell; V. P., D. H. Smith, Bath; Secretary-Treasurer, W. W. Smith, Avoca; Censors, Frank Shaut, Addison; T. H. Pawling, Avoca; B. R. Wakeman, Hornell.

